# Anticancer Effects of Valproic Acid via Regulation of Epigenetic Mechanisms in Non-small-cell Lung Cancer A549 Cell Line 

**DOI:** 10.22037/ijpr.2019.111945.13442

**Published:** 2021

**Authors:** Hadi Kalantar, Mohsen Rashidi, Mojtaba Kalantar, Mahmoud Tavallaei, Sayed Mostafa Hosseini

**Affiliations:** a *Toxicology Research Center, Medical Basic Sciences Research Institute, Ahvaz Jundishapur University of Medical Sciences, Ahvaz, Iran. *; b *Department of Pharmacology, Faculty of Medicine, Mazandaran University of Medical Sciences, Sari, Iran. *; c *Shoushtar Faculty of Medical Sciences, Shoushtar, Iran.*; d *Human Genetics Research Center, Baqiyatallah University of Medical Sciences, Tehran, Iran. *

**Keywords:** Valproic acid, Lung cancer, Metastases, Nm23H1, CD44v6

## Abstract

Epigenetic mechanisms are the most important factors contributing to both the development and metastasis of cancer cells. We aimed to scrutinize the role of epigenetic alternations of genes involved in cancer metastasis, including *CD44v6 *(metastasis indicator) and *Nm23-H1* (a novel tumor suppressor), in the A549 lung cancer cell line. The A549 cells were cultured in the DMEM medium. Valproic acid (VPA) was used as a histone deacetylase inhibitor. Caspase-3 activity was assessed by adding DEVD-pNA substrate to the cell lysate. Gene expression was determined by real-time PCR. Finally, protein expression was assessed by western blot. The results showed that VA significantly decreased the expression of the *CD44v6 *gene and its protein level. This was further accompanied by lower expressions of *MMP-2* and *MMP-9* genes. On the other hand, the expression of *Nm23-H1* and its protein were significantly increased in the cells accompanied by higher activity of caspase-3 (*P ˂ *0.05). Our results showed that epigenetic regulation of *CD44v6*, *Nm23-H1*, *MMP-2,* and *MMP-9 *might be involved in the pathogenesis and metastasis of lung cancer. Therefore, the use of histone deacetylase inhibitors can be effective in the suppression of metastases and the treatment of these tumors.

## Introduction

Lung carcinoma represents high metastatic capacity, as well as a high rate of morbidity and mortality. These tumors comprise 17% of all human cancers per year (1). The predominant form of lung cancer is non-small cell lung cancer (NSCLC), accounting for about 85% of all lung cancer cases (2). Metastasis is frequently encountered in NSCLC reported in about 30–40% of the patients (3). Multiple mechanisms and a variety of genetic interactions are involved in this process. In this regard, proto-oncogenes and tumor suppressors are master regulators in cancer pathogenesis and metastasis. *Nm23-H1*, also known as NDPK-A or NME1, has been identified tumor suppressor gene inhibiting metastasis in various cancers (4). Nm23-H1 has been down-regulated in highly metastatic tumors as mice lacking the *Nm23-M1* gene developed lung cancer more frequently than wild-type controls (5). The mechanisms of antitumor activities of *Nm23-H1* are yet to be divulged. Nevertheless, modulation of multiple growth factors and matrix metalloproteinases (MMPs) may be involved (6).

The expression of *Nm23-H1* is highly regulated by epigenetic factors including histone deacetylases (HDAC). Valproic acid (VPA) is a promising and novel HDAC inhibitor and anti-cancer agent. The administration of VPA has been associated with the downregulation of *Nm23-H1* and reduced proliferative capacity of breast cancer cells (7). 

Metastasis is a complex phenomenon involving the migration of cells through expressing transmembrane cell adhesion molecules (8). CD44 is a multifunctional transmembrane glycoprotein participating in cell–cell and cell–matrix interactions during tumorigenesis, angiogenesis, tumor growth, and metastasis (9). Particular attention has been given to *CD44v6 *which is involved in cell–cell and cell–matrix interactions during tumorigenesis (10). Studies have demonstrated the critical role of this protein in the motility and adhesion of cancerous cells to the base membranes (11). It has recently been noted that concomitant inhibition of *CD44v6 *and matrix metalloproteinase-9 (*MMP-9*) lowers migration of neoplastic cells (12). 

We here assessed the effects of VPA (as a new HDAC inhibitor) on the expression of *Nm23-H1* and *CD44v6 *(as a novel tumor suppressor and a metastasis marker, respectively) in the A549 cell line of NSCLC. 

## Experimental

*Ethics Statement*

The study was approved by the Baqiyatallah University of Medical Sciences (code: IR. BMSU.REC.1395.1203).

*Cell Culture*

The A549 human NSCLC cell line was purchased from Pasture Institute of Iran (Tehran, Iran). The cells were grown in Dulbecco’s modified Eagle’s medium (DMEM /F-12 with GlutaMAX, Gibco, 10565018; Thermo Fisher Scientific Inc., Waltham, MA), which was supplemented with 10% fetal bovine serum (Gemini Bio-Products, West Sacramento, CA), 100 U/mL penicillin G, and 10 mg/mL streptomycin (Invitrogen, Carlsbad, CA). The culture bottles were incubated at 37 °C and 5% CO2. The culture medium was refreshed every 3–4 days. The cells were detached from the old culture using 0.25 mg/mL trypsin/EDTA (Invitrogen). 

*MTT assay*

The viability of the A549 cells treated with VPA was assessed using the standard 3-(4, 5-dimethylthiazol-2-yl)-2, 5-diphenyltetrazolium bromide (MTT) assay. Briefly, the cells were seeded at 104/cells per well in a 96-well plate and incubated overnight at 37 °C and 5% CO2 for 24 h. After refreshing the culture medium, the cells were treated with various concentrations of VPA (0–16 mM) for either 24 h, 48 h, or 72 h. Then, MTT reagent was added at the final concentration of 500 μg/mL to each well, and the plate was further incubated at 37 °C for 4 h in the dark. Finally, the supernatant was discarded and 150 μL DMSO was added to each well. The absorbance was measured at 570 nm with a reference filter of 630 nm using the Synergy H1 Hybrid Multi-Mode Reader (BioTek Instruments, Inc., Winooski, VT). The percentage of alive cells in the presence of VPA was determined respective to the control cells grown in the absence of VPA (13). The IC_50_ was determined using GraphPad Prism software version 7.03. 

*Measurement of caspase-3 activity*

To measure caspase-3 activity, a caspase-3 substrate (DEVD-pNA, BioVision, Inc., Milpitas, CA) was utilized. For this purpose, the cells treated with various doses of VPA were lysed using chilled cell lysis buffer (BioVision, Inc.). After measuring the protein concentration of the cell lysate using the bicinchoninic acid (BCA) method, equal volumes of protein (100 μg) were diluted to a total volume of 50 μL and mixed with 50 μL of 2X reaction buffer (BioVision, Inc.). The DEVD-pNA substrate was then added to the diluted cell lysates and incubated at 37 °C for 2 h (14). Finally, the absorbance of the released pNA was measured at 405 nm.

*RNA extraction and real-time polymerase chain reaction (RT-PCR)*

According to the manufacturer’s guidelines, the total RNA was extracted using RNX Plus reagent (Cinnagen, Iran). The quality and quantity of the extracted RNA were assessed by measuring the absorbance ratios of 260/230 nm and 260/280 nm, respectively (NanoDrop spectrophotometer, BioTek, USA). The extracted RNA was further purified using DNase I (Thermo Fisher Scientific Inc.) digestion. Complementary DNA (cDNA) was synthesized using the PrimeScript RT reagent kit (Thermo Fisher Scientific Inc.). Real-time polymerase chain reaction (RT-PCR) was used to determine gene expressions using SYBR Green Master Mix (Parstoos, Iran) and ABI Step One Real-Time PCR System (Applied Biosystems, Foster City, CA). The primers sequences for the genes have been shown in Table 1. All reactions were done in triplicate under the following conditions: 10 min at 95 °C (initial denaturation) followed by 30 cycles as 15 s in 95 °C (denaturation), and 30 s in 60 °C (annealing) and 72 °C (extension). The melting curve analysis was performed within 60 °C to 95 °C. The relative gene expression was calculated using 2–ΔΔCt method (15).

*Western blotting*

The cells seeded in 6-well plates were scratched and transferred to a microtube. For protein extraction, the cells were suspended in RIPA lysis buffer (Santa Cruz Biotechnology, Inc., Dallas, TX) containing protease inhibitor (Sigma-Aldrich, St. Louis, MO). After that, the cells were sonicated and centrifuged for 10 min at 14,000 rpm and 4 °C. The protein content of the supernatant was evaluated using the BCA method. For each group, 50 µg protein was electrophoresed on 10% SDS-PAGE. The protein bands were then transferred to nitrocellulose membranes (Millipore) using a semi-dry transfer membrane system (Cleaver Scientific Ltd, Warwickshire, United Kingdom). The blocking was performed using 5% skim milk in TBS buffer (20 mM Tris–HCl, 500 mM NaCl, pH 7.4). Mouse anti-human *NM23-H1 *(Santa Cruz Biotechnology, Inc.) and mouse anti-human *CD44v6 *(Abcam, Cambridge, UK) antibodies were diluted (1:1000) in TBST buffer (20 mM Tris–HCl, 500 mM NaCl, 0.5% Tween 20, pH 7.4). The membrane was incubated with the primary antibodies overnight at 4 °C. The secondary HRP-conjugated anti-mouse antibody (Santa Cruz Biotechnology, Inc.) diluted in TBST buffer (1:10000) was then added to the membrane and incubated for 1 h at 37 °C. Finally, enhanced chemiluminescence (Thermo Fisher Scientific Inc.) was used followed by exposure to radiographic film to detect secondary antibody binding.

*Statistical Analysis*

Statistical analysis was performed in SPSS 19. All the tests were done in triplicate. Data were expressed as mean ± SD. One-way ANOVA followed by post hoc Tukey test was used for comparisons between groups. *P* < 0.05 was considered as the statistical significance threshold. 

## Results

*Cytotoxicity of VPA against A549 Cells*

**Figure 1:**
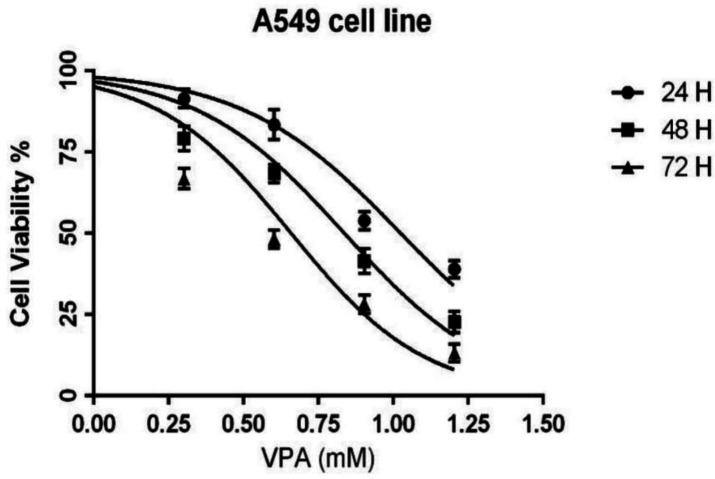
The effect of VPA (0-16 Mm) on the cell viability of A549 cells after 24, 48 and 72 h incubation. Results are expressed as means ± SEM, n = 3

The cells were exposed to different concentrations (0-16 mM) of AVP for either 24, 48 or 72 h. The MTT results showed that VPA inhibited the growth of A549 cells in a concentration- and time-dependent manner (Figure 1). The IC_50_ values of VPA for A549 cells were 10.5, 6.8 and 4.5 mM in 24, 48 and 72 h incubations, respectively.

*VPA promotes caspase-3 activity in A549 cells*

**Figure 2:**
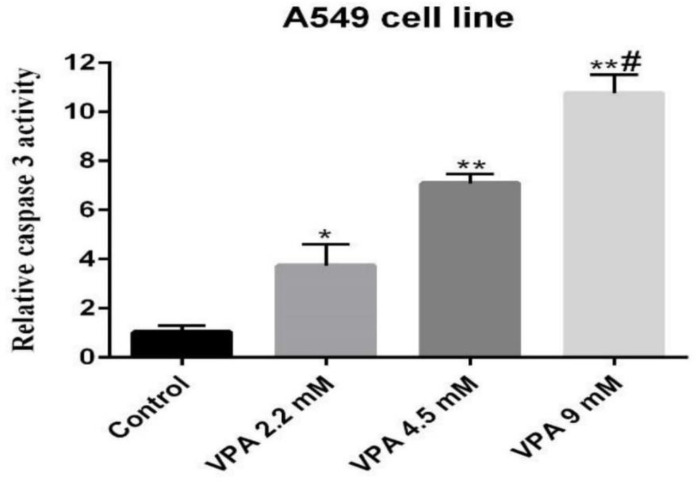
Relative caspase 3 activity was determined in 549 cell line treated with 2.2, 4.5 and 9 mM of VPA for 72 h. *(*P ˂ *0.01), **(*P ˂ *0.001) compared to control cells, ^#^(*P ˂ *0.001) compared to 2.2 and 4.5 mM of VPA

Caspase-3 activity, an early marker of apoptosis, was determined in A549 cells exposed to VPA. Accordingly, the cells exposed to 9 mM of VPA for 72 h showed significantly higher caspase-3 activity respective to other concentrations and periods (*P ˂ *0.001, Figure 2).

*VPA suppressed MMP-2 and MMP-9 genes expression*

**Figure 3:**
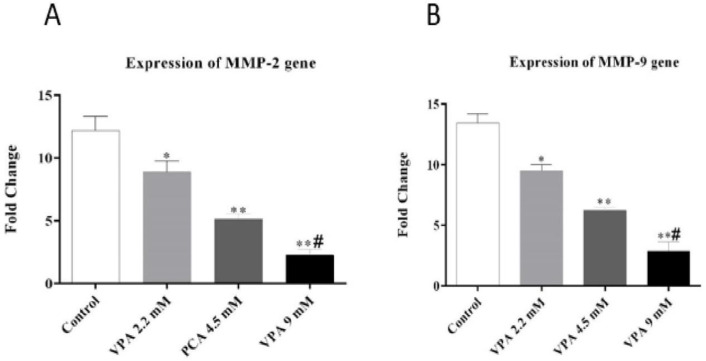
Effect of different concentrations of VPA on the expression of *MMP-2* and *MMP-9 *gene after 72 h incubation. ^*^(*P ˂ *0.01), ^**^(*P ˂ *0.001) compared to control cells, ^#^(*P ˂ *0.001) compared to 2.2 and 4.5 mM of VPA

 As shown in Figures 3A and 3B, VPA significantly suppressed the expression of *MMP-2* and *MMP-9 *in a dose-dependent manner (*P ˂ *0.01 and* P ˂ *0.001). 

*VPA promoted Nm23H-1 and alleviated CD44v6 expression in A549 cells*

**Figure 4:**
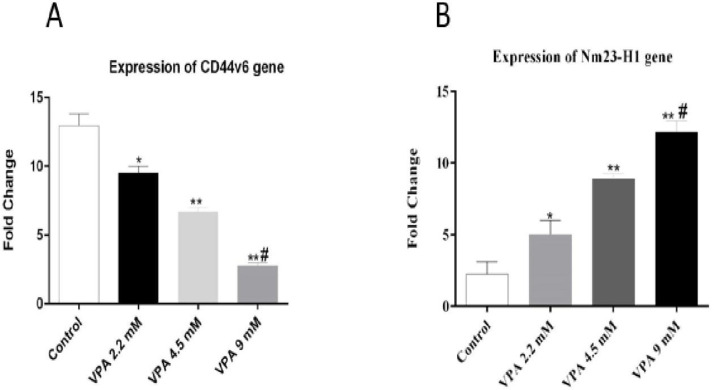
Effect of different concentrations of VPA on the expression of *Nm23-H1 *and *CD44v6 *gene after 72 h incubation.^ *^(*P ˂ *0.01), ^**^(*P ˂ *0.001), compared to control cells, ^#^(*P ˂ *0.001) compared to 2.2 and 4.5 mM of VPA

**Figure 5:**
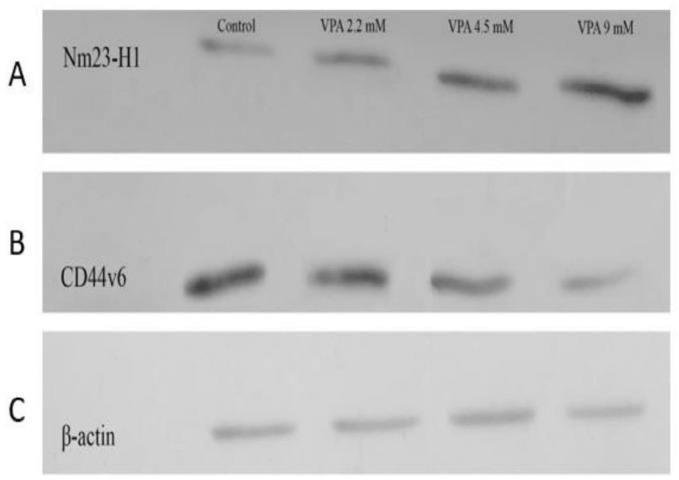
Effect of VPA on the expression of *Nm23-H1 *and *CD44v6 *protein in A549 cell line. VPA increased and reduced the expression of Nm23H1 and *CD44v6 *protein in A549 after 72 h, respectively

The expressions of *Nm23-H1* and *CD44v6 *andtheir protein levels, were investigated in A549 cells treated with VPA for 72 h. VPA treatment significantly alleviated the expression of *CD44v6 *gene (Figure 4A, *P* < 0.001) and protein (Figure 5A) in a dose-dependent way (*P* < 0.001). On the other hand, the cells exposed to 9 mM VPA represented significantly upregulated *Nm23-H1* gene (Figure 4B, *P *< 0.001) and protein (Figure 5B).

## Discussion

In this study, we assessed the effects of VPA, a histone deacetylase inhibitor, to determine its effects on the expression of genes involved in tumor metastasis in the A549 lung cancer cell line. Histone acetylation is one of the most important epigenetic mechanisms modulating gene expression. We observed here that VPA decreased the viability of A549 cells upon 72 h incubation in a dose-dependent manner. Inline, a dose-dependent increase was observed at both gene and protein levels of *Nm23-H1*, a tumor suppressor in the cancerous cells. On the other hand, the expression of metastatic tumor indicators, *CD44v6*, *MMP-2*, and *MMP-9 *decreased in A549 cells exposed to VPA. These changes further were accompanied by increased caspase-3 activity in VPA treated A549 cells. 

*Nm23-H1 *is a multifunctional protein with nucleoside diphosphate kinase, histidine kinase, and DNase activities (16). Studies have shown the antineoplastic activity of HDACs in hematologic and solid malignancies. Scientific evidence also supports the key role of HDACs in down-regulating of genes involved in tumor metastasis and invasion both *in-vitro* and *in-vivo* (17, 18). HDAC inhibitors can suppress tumorigenesis by halting cancer cells migration, invasion, and growth, as well as by inducing apoptosis (19, 20). In this study, we showed that VPA as an HDAC inhibitor, induced the expression of *Nm23-H1*, a tumor suppressor downregulated in highly metastatic cancers (4). *Nm23-H1 *upregulation can induce DNA damage and subsequently genomic instability (21). Based on our findings and the effects of VPA on *Nm23-H1*, epigenetic alternations of this gene in A459 cancer cells may also be a mechanism involved in the metastatic behavior of lung cancer cells. Therefore, applying HDACs inhibitors, particularly VPA, may provide a therapeutic option in this type of cancer. 

HDACs also can induce apoptosis in cancer cells (19, 20). Accordingly, it has been shown that VPA-induced apoptosis may involve an increase in acetylation of histones and tubulin, a study conducted in a gastric cancer cell line (22). In our study, the apoptotic effect of VPA on the lung cancer cell line A549 was accompanied by an increase in caspase-3 activity. This result agreed with a previous study that reported the up-regulatory effect of VPA on caspase-3 (23). 

*CD44* is a cell-surface glycoprotein involved in cell–cell and cell–matrix adhesion, cell migration, and metastasis. Among all *CD44* isoforms, *CD44v6 *harboring a mutation in exon 11 plays an important role in enhancing the adhesive ability of tumor cells (24). The adhesion of cancer cells to the basal epidermal components such as collagen, integrin, and fibronectin is mediated by *CD44v6*. An evidence-based report showed that an increase in the expression of *CD44v6 *altered the physicochemical properties of tumor cells and increased their metastatic potential (25). *CD44v6 *the We showed the inhibitory effects of VPA on *CD44v6 *at both gene and protein levels in the A459 cell line in the present study. This observation indicates a potential inhibitory impact for VPA on tumorigenesis in lung cancer. 

Our results also revealed an inhibitory effect for VPA on the expression of *MMP-2* and *MMP-9 *genes in the A459 cell. Type I collagenases such as *MMP-2* and *MMP-9 *participate in cancer growth and invasion by degrading extracellular matrix (ECM) (26). *MMP-2* and *MMP-9 *are zinc-dependent ECM degrading enzymes involved in the metastatic activity of tumor cells (27). According to the effects of VPA on the expression of these genes in A459 cells observed here, HDACs and epigenetic mechanisms may be involved in lung cancer progression. Therefore, HDACs inhibitors such as VPA can provide a viable therapeutic agent in these cancers. 

In the present study, the up-regulation of *Nm23-H1* upregulation was seen in concomitant with the down-regulation of *MMP-9*. This phenomenon can promote a potent anti-metastatic effect on cancer cells. The interaction between *Nm23-H1 *and *MMP-9 *is controversial. *Nm23-H1* has been shown to increase *MMP-9 *gene expression and its gelatinolytic activity (28). In another report, however, *Nm23-H1* did not modify *MMP-9 *expression (29). Moreover, *Nm23-H1* upregulation has been related to *MMP-9 *suppression in yet another report (30). In our study, increased *Nm23-H1* expression was seen in parallel to decreased expression of *CD44v6*, *MMP-2*, and *MMP-9*. This event, along with elevated caspase-3 activity, can finally result in apoptosis in A549 human lung cancer cells. 

In conclusion, our study demonstrated the antitumor activity of VPA against the A549 lung cancer cell line. One possible mechanism may be the Inhibition of HDAC, which increased *Nm23-H1* expression. Furthermore, VPA-treated A549 cells showed decreased expression of *CD44v6*, *MMP-2*, and *MMP-9 *considered as metastasis indicators in cancer. The underlying inhibitory mechanisms of VPA on tumor cells and its potential therapeutic role in cancer are yet to be investigated.

**Table 1:** Primer sequences were used in RT-PCR

**Gene**	**F/R**	**Primer sequences (5'3')**
*Nm23H1*	Forward	TTAATCAGATGGTCGGGGAT
	Reverse	GATCTATGAATGACAGGAGG
*CD44V6*	Forward	GTCGATGCTAGCTAGCCGTAGCATG
	Reverse	CGAGCTAGTCGTAGTCGATCGATCG
*MMP2*	Forward	TCTCCTGACATTGACCTTGGC
	Reverse	CAAGGTGCTGGCTGAGTAGATC
*MMP9*	Forward	CCTTGTGCTCTTCCCTGGAG
	Reverse	GGCCCCAGAGATTTCGACTC
*GAPDH*	Forward	AATCCCATCACCATCTTCCA
	Reverse	TGGACTCCACGACGTACTCA
